# Vulnerability to low-dose combination of irinotecan and niraparib in ATM-mutated colorectal cancer

**DOI:** 10.1186/s13046-020-01811-8

**Published:** 2021-01-06

**Authors:** Pietro Paolo Vitiello, Giulia Martini, Luigi Mele, Emilio Francesco Giunta, Vincenzo De Falco, Davide Ciardiello, Valentina Belli, Claudia Cardone, Nunzia Matrone, Luca Poliero, Virginia Tirino, Stefania Napolitano, Carminia Della Corte, Francesco Selvaggi, Gianpaolo Papaccio, Teresa Troiani, Floriana Morgillo, Vincenzo Desiderio, Fortunato Ciardiello, Erika Martinelli

**Affiliations:** 1 Department of Precision Medicine, Medical Oncology, Università degli Studi della Campania Luigi Vanvitelli, Naples, Campania Italy; 2 Department of Experimental Medicine, Università degli Studi della Campania Luigi Vanvitelli, Naples, Campania Italy; 3 Department of Medical, Surgical, General and oncology surgery, Neurologic, Metabolic and Ageing Sciences, Università degli Studi della Campania Luigi Vanvitelli, Naples, Campania Italy

**Keywords:** Colorectal cancer, DNA damage response, Homologous recombination, Combination treatment, Chemopotentiation, Synergism, Irinotecan, PARP inhibitors

## Abstract

**Background:**

Despite the advancements in new therapies for colorectal cancer (CRC), chemotherapy still constitutes the mainstay of the medical treatment. For this reason, new strategies to increase the efficacy of chemotherapy are desirable. Poly-ADP-Ribose Polymerase inhibitors (PARPi) have shown to increase the activity of DNA damaging chemotherapeutics used in the treatment of CRC, however previous clinical trials failed to validate these results and pointed out dose-limiting toxicities that hamper the use of such combinations in unselected CRC patients. Nevertheless, in these studies little attention was paid to the mutational status of homologous recombination repair (HRR) genes.

**Methods:**

We tested the combination of the PARPi niraparib with either 5-fluorouracil, oxaliplatin or irinotecan (SN38) in a panel of 12 molecularly annotated CRC cell lines, encompassing the 4 consensus molecular subtypes (CMSs). Synergism was calculated using the Chou-Talalay method for drug interaction. A correlation between synergism and genetic alterations in genes involved in homologous recombination (HR) repair was performed. We used clonogenic assays, mice xenograft models and patient-derived 3D spheroids to validate the results. The induction of DNA damage was studied by immunofluorescence.

**Results:**

We showed that human CRC cell lines, as well as patient-derived 3D spheroids, harboring pathogenic ATM mutations are significantly vulnerable to PARPi/chemotherapy combination at low doses, regardless of consensus molecular subtypes (CMS) and microsatellite status. The strongest synergism was shown for the combination of niraparib with irinotecan, and the presence of ATM mutations was associated to a delay in the resolution of double strand breaks (DSBs) through HRR and DNA damage persistence.

**Conclusions:**

This work demonstrates that a numerically relevant subset of CRCs carrying heterozygous ATM mutations may benefit from the combination treatment with low doses of niraparib and irinotecan, suggesting a new potential approach in the treatment of ATM-mutated CRC, that deserves to be prospectively validated in clinical trials.

## Background

Colorectal cancer (CRC) is the third most frequent malignancy and the second leading cause of cancer death worldwide, accounting for more than 1.8 million new cases and 880,000 deaths in 2018 [1].

Despite the increase in therapeutic options for the treatment of metastatic CRC (mCRC), cytotoxic chemotherapy using fluoropyrimidines, oxaliplatin, and irinotecan still constitutes the mainstay of the treatment [2]. These genotoxic drugs function by inducing direct or indirect DNA damage, which lead to cell cycle block and cell death. Nevertheless, DNA damage is recognized by different DNA repair pathways such as mismatch repair (MMR), nucleotide excision repair (NER), and homologous recombination repair (HRR) [3]. All these mechanisms of DNA damage response (DDR) are crucial for the survival of normal cells and are often deregulated in cancer, allowing for the accumulation of mutations that are ultimately associated to cancer progression and therapeutic resistance [4]. For these reasons, blocking DDR has been widely investigated in the context of cancer treatments as a strategy to potentiate radiotherapy/chemotherapy-induced DNA damage and overcome drug resistance. Indeed, several compounds that interfere with the different mechanisms of DNA repair are already available or in advanced clinical testing [5]. Among these compounds, Poly-ADP-Ribose Polymerase inhibitors (PARPi) were the first to be approved in clinical practice and exhibit a strong activity in ovarian and in other cancers characterized by a functional impairment of HRR known as homologous recombination deficiency (HRD) [6–8]. In the last years, several biomarkers were used to predict HRD and sensitivity to PARP inhibitors, most of them focused on finding genetic alteration in HRR-associated genes (mainly BRCA1/2, but also ATM, PALB2 and others) and/or on analyzing the *genomic scars* associated to HRD (loss of heterozygosity, large scale transitions, subchromosomal allelic imbalance) [9]. Moreover, mutational signature single base substitution 3 (SBS3) has been associated to HRD and correlates with PARPi sensitivity [10].

Initial preclinical data reported PARPi efficacy also in CRC cell lines, apparently increased in case of microsatellite instability (MSI). Notably, in the largest analyzed preclinical cohort, including more than 100 human CRC cell lines, no clear association was evidenced between olaparib sensitivity (13% of MSS cell lines screened) and either mutations in HR genes or mutational signatures [11]. However, a phase II clinical trial with olaparib did not show any benefit in chemorefractory mCRC patients [12, 13]. In addition, several preclinical reports evidenced promising synergism of PARP inhibitors in combination with oxaliplatin or irinotecan in CRC, independently from microsatellite status [14–16]. However, similarly to previous cases, a phase Ib clinical trial failed to identify any benefits for the combination of the PARP inhibitor olaparib and irinotecan in chemorefractory mCRC [17]. Very disappointedly, this study evidenced a high-grade hematological toxicity that led to a significant dose reduction, which probably hampered the efficacy in this unselected population [17]. These results might be explained by the low prevalence of biallelic loss in genes involved in homologous recombination in CRC, which is less than 3% versus more than 50% in ovarian cancer [18]. Nevertheless, 26% of mCRC patients from the large MSK IMPACT database exhibit at least one mutation in HRR genes, the most frequent being ATM (8%) and BRCA2 (8%) [19]. Taking this into account, in this study we hypothesized that HRR genes mutations might represent a vulnerability to the combination of PARPi/chemotherapy in CRC. Thus, we have systematically evaluated the synergism between the PARPi niraparib and three genotoxic agents approved for mCRC in a panel of 12 human CRC cell lines representative of the main molecular subtypes, showing that irinotecan is the best candidate for combination therapy. Interestingly, in our work we have identified ATM mutations as a common genetic background associated with niraparib/ irinotecan combination efficacy. We further confirmed that a low dose combination is very effective in an in vivo model and in primary 3D cultures obtained from fresh CRC surgical specimens, which are mutated in ATM or in its downstream pathway. Moreover, we showed that a malfunction of HRR due to heterozygous mutations in ATM is responsible for such effects.

## Methods

### Research resource identifiers (RRIDs)

In order to support rigor and transparency in this publication, key resources such as Antibodies, Model Organisms, Cell Lines, and Softwares have been matched to their unique identified from the RRID portal (https://scicrunch.org/resources).

### Drugs and chemicals

Niraparib (Cat # S2741) and SN38 (Cat # S4908) were purchased from Selleckchem. Both drugs were dissolved in sterile DMSO at 10 mmol/L stock solution concentration and stored in aliquots at − 20 °C. Methylcellulose (Methocel, Cat #64632) was purchased from Sigma-Aldrich. Irinotecan, oxaliplatin and 5-fluorouracil were kindly provided by the hospital pharmacy service of the Oncology Unit of University of Campania. Working concentrations were diluted in culture medium just before each experiment. SN38 was used for in vitro experiments as the active metabolite of irinotecan, while irinotecan was used for mice xenograft experiments. Niraparib for animal studies was resuspended in 0.5% w/v methylcellulose, while irinotecan was resuspended in sterile saline for infusions.

### Cell line cultures and mutational profiles of cell lines

Human HCT15 (RRID:CVCL_0292; ATCC Cat# CCL-225), LOVO (RRID:CVCL_0399; ATCC Cat# CCL-229), SW1116 (RRID:CVCL_0544; ATCC Cat# CCL-233), LS1034 (RRID:CVCL_1382; ATCC Cat# CRL-2158), SW403 (RRID:CVCL_0545; ATCC Cat# CCL-230), SW948 (RRID:CVCL_0632; ATCC Cat# CCL-237), CACO2 (RRID:CVCL_0025; ATCC Cat# HTB-37) and WIDR (RRID:CVCL_2760; ATCC Cat# CCL-218) authenticated colorectal cancer cell lines were obtained from the American Type Culture Collection (ATCC). The human SW48 (RRID:CVCL_1724; Cat# HTL99020), HCT116 (RRID:CVCL_0291; Cat# HTL95025), SW480 (RRID:CVCL_0546; Cat# HTL99017) cell lines were obtained from Istituto di Ricovero e Cura a Carattere Scientifico (IRCCS) “Azienda Ospedaliera Universitaria San Martino-Istituto Nazionale per la Ricerca sul Cancro, Genova,” Italy. LIM1215 (RRID:CVCL_2574) CRC cell line was obtained from Dr. F. Di Nicolantonio (Candiolo National Cancer Institute, Candiolo, Italy) and authenticated by IRCCS “Azienda Ospedaliera Universitaria San Martino-IST Istituto Nazionale per la Ricerca sul Cancro, Genova,” Italy. Cells were grown in RPMI- 1640, DMEM/F12, EMEM or McCoy medium (Lonza), supplemented with 10% FBS and 1% penicillin/streptomycin, in a humidified incubator with 5% of carbon dioxide (CO_2_) and 95% air at 37 °C and were routinely screened for the presence of mycoplasma (Mycoplasma Detection Kit; Roche Diagnostics). Microsatellite status and transcriptional profiling according to the consensus molecular subtypes (CMS) were obtained from the work of Sveen and colleagues [20]. Mutational profiles in 29 relevant homologous recombination repair genes [18] were obtained from cBio-portal [21, 22] using the Cancer Cell Line Encyclopedia (CCLE) dataset [23, 24] [last accessed June 20th 2020]. Functional prediction for ATM and BRCA2 mutations were obtained using FATHMM algorithm integrated in COSMIC [25] or Leiden Open Variants Database (LOVD) [26] [last accessed July 1st 2020].

### Proliferation and colony assays

Cell proliferation was analyzed by the MTT assay (Sigma-Aldrich), according to manufacturer’s instructions. Briefly, for each cell line 2–10 × 10^3^ cells/well were plated in 48 multiwell plates. After 24 h, cells were treated with different concentrations of niraparib, 5-fluorouracil, oxaliplatin, SN38 or niraparib/chemotherapy combination for 96 h. The IC_50_ values were determined by using the CompuSyn 1.0 and plotted in dose response curves using Graphpad Prism 8.0 (RRID: SCR_002798). Results represent the median of the three experiments, each performed in triplicate. Combinations were performed according to IC_50_ ratio, as described by the Chou-Talalay model [27], and combination index was obtained using CompuSyn 1.0 (Combosyn Inc.).

Colony formation assay was performed to evaluate the long-term proliferative potential of cell lines following treatments with niraparib, SN38 or their combination at different concentration ratios (100:1; 50:1) and dose levels. For each experiment, 3–15 × 10^3^ cells/well were seeded in 6-well plates and incubated with the drugs in serum-containing medium for 24 h. The medium was then replaced with fresh culture medium every 3 days. After 14 days, cells were fixed with 4% paraformaldehyde at room temperature (RT) for 15 min, stained with 0.1% crystal violet and counted using ImageJ (RRID:SCR_003070). Results represent the median of at least two separate experiments, each performed in duplicate.

### Mice xenografts

Four- to six-week-old female athymic nude mice (NU-Foxn1^nu^, IMSR Cat# CRL:194, RRID:IMSR_CRL:194) were purchased from the Charles River Laboratories. LS1034, CACO2, HCT116, WIDR and SW48 human colorectal cancer cell lines were used. A total of 3–5 × 10^6^ cells was resuspended in 200 μL of Matrigel (BD Biosciences) and PBS (1:1) and implanted subcutaneously into the right flank of 20 mice for each cell line. Once tumors reached a volume of 75–100 mm^3^, mice were randomized to 4 arms each of 5 mice: control arm to receive vehicle alone (0.5% methylcellulose per os using oral gavage, 5 days a week + PBS intraperitoneally, 2 days a week), irinotecan arm (10 mg/kg intraperitoneally, 2 days a week), niraparib arm (50 mg/kg per os using oral gavage, 5 consecutive days a week), and their combination. Treatment was continued for a total of 4 weekly cycles. Tumor measurements were performed twice a week using a caliper, tumor volumes were calculated using the formula: V = (W^2^ × L)/2. Relative tumor volume (RTV) was calculated for each tumor relative to day 1 of treatment. After treatment end, mice were followed up for survival analysis up to 100 days. Mice were euthanized in case of tumor volume > 2000 mm^3^, tumor ulceration or onset of distress.

### Immunofluorescence

25 × 10^3^ cells/well were seeded on a coverslip in a 12-multiwell and treated for 24 h with 100 nmol/L niraparib, 1 nmol/L SN38 or their combination. After 24 h, cells were washed in PBS and fresh culture medium without drugs was added in order to allow DNA damage recovery. After additional 24 h, cells were washed in PBS, fixed with 4% paraformaldehyde (PFA) solution and permeabilized with 0.1% TRITON -X/PBS solution, then blocking was performed in 1% BSA for 1 h at RT. Cells were incubated with rabbit primary anti-RAD51 antibody (Abcam Cat# ab133534, RRID:AB_2722613) and mouse anti-phospho-Histone-H2AX antibody (Millipore Cat# 05–636-I, RRID:AB_2755003) in PBS for 90 min. Secondary goat anti-rabbit TRITC-conjugated (Abcam Cat# ab6718, RRID: AB_955551) and donkey anti-mouse FITC-conjugated (Abcam Cat# ab150105, RRID:AB_2732856) antibodies were added after a PBS wash in the same conditions. Cells were incubated in a 1:500 solution of 10 mg/mL Hoechst (Invitrogen) in PBS for 10 min in the dark. Images were collected under a fluorescence microscope (EVOS FL Cell Imaging System, Thermo Scientific, Rockford, USA). Each experiment was performed in quadruplicate and at least 100 nuclei were considered in each replicate. ImageJ (Fiji plugin, RRID:SCR_002285) was used to generate merged images and quantify colocalization puncta.

### Cell cycle analysis

50 × 10^3^ cells/well were seeded in six-well plates using FBS-containing medium. After 24 h cells were serum starved for 24 h and then treated for 24 h in serum-containing medium with 100 nmol/L niraparib, 1 nmol/L SN38 or their combination. Cell pellets were harvested in phosphate-buffered saline (PBS) containing 2 mM EDTA, washed once with PBS, fixed in iced ethanol 70%, washed with PBS and incubated with 25 μg/ml PI plus RNase (Invitrogen) 1 mg/ml for 120 min at 4 °C in the dark. Stained nuclei were analysed with FACS Aria III (Becton and Dickinson, Mountain View, CA, USA), and data analysed using ModFit 2.0 cell cycle analysis software (ModFit LT, Verity Software House, Topsham, UK. RRID:SCR_016106).

### 3D primary cell cultures

Fresh tissue specimens, derived from primary or metastatic colorectal cancers from patients enrolled in the I-CURE project (Regione Campania), were transported to the laboratory within 2 h from surgical harvesting. The tissues were weighed, washed twice with phosphate buffered solution (PBS) and cut in fragments. Briefly, tumor fragments were incubated with a shake with digestion medium (DMEM F-12, Sigma-Aldrich) supplemented with 2% Penicillin/Streptomicin, 10X Amphotericin, 2X Collagenase and Hyaluronidase for up 6 h in a 37 °C. All undigested fragments and debris were filtered through a cell strainer (BD-Falcon) after digestion followed by centrifugation for 5 min at 300 rcf. The supernatant was removed, and the pellet was washed with PBS and then centrifuged as described above. The pellet was further re-suspended in ice-cold 1:1 mixture of growth medium and Matrigel (BD-Falcon) and then seeded in 24 well-plates (Corning). The matrigel droplets were polymerized for 30 min a 37 °C and growth medium was added after polymerization. All patient-derived tumor spheroids originated from primary colonic cancer surgical samples from untreated (chemo-naïve) patients, with the exception of IC-001 that was generated from an abdominal wall metastasis from a patient previously treated with 5FU, oxaliplatin and irinotecan. For drug screening experiments, 10–20 × 10^3^ tumor spheroids/well were plated as described in 24 multiwell plates; after 48 h, spheroids were treated with 1000 nM niraparib, 10 nM SN38 or their combinations. Growth inhibition was performed using MTT assay (Sigma-Aldrich), normalized on untreated control, after 14 days of treatment. Matrigel was degraded using Cell Recovery Solution (BD-Falcon) according to the manufacturer’s procedures and pellets collected for absorbance detection. These experiments were performed in triplicates.

### Genomic profiling of patients’ tumor specimens

FoundationOne® (F1CDx) was performed in a single site at Foundation Medicine, using adequate tissue specimens from FFPE blocks to provide a minimum yield of 55 ng of genomic DNA to ensure enough DNA for quality control (QC) and to proceed with library construction. In total, the assay detects alterations in 324 genes. Using the Illumina HiSeq 4000 platform, hybrid capture-selected libraries are sequenced to high uniform depth (targeting >500X median coverage with > 99% of exons at coverage > 100X). Additionally, genomic signatures including MSI and TMB are reported. To determine MSI status, 95 intronic homopolymer repeat loci (10–20 bp long in the human reference genome) with adequate coverage on F1CDx Assays are analysed for length variability and compiled into an overall MSI score via principal components analysis. Each sample is assigned a qualitative status of microsatellite instable (MSI) or microsatellite stable (MSS). Tumor Mutational Burden (TMB) is a quantitative index of the number of mutations present in the cancer genome. TMB is measured by counting all synonymous and non-synonymous variants present at 5% allele frequency or greater and filtering out potential germline variants according to published databases of known germline polymorphisms including Single Nucleotide Polymorphism database and Exome Aggregation Consortium. The resulting mutation number is then divided by the coding region corresponding to the number of total variants counted or 793 kb. The derived number is communicated as mutations per Mb unit (mut/Mb): low TMB for 1–5 mut/Mb, intermediate TMB for 6–19 mut/Mb, high TMB for ≥20 mut/Mb). Approved results are annotated by automated software with CDx relevant information and are merged with patient demographic information.

### Statistical analyses

All statistical analyses were performed using Graphpad Prism 8.0 software (RRID: SCR_002798). Distribution of IC_50_ or combination index values according to molecular features was calculated using Wilcoxon-Mann-Whitney test. Quantitative in vitro and in vivo data were reported as mean ± standard deviation (SD). Results were compared by analysis of variance (ANOVA), and a *p* value < 0.05 was considered statistically significant. Survival analysis was carried out visually by means of Kaplan-Meier curves, while the difference in survival in each arm was calculated using the log-rank test.

## Results

### Activity of niraparib, 5-fluorouracil, oxaliplatin, and SN38 or niraparib/chemotherapy combinations on human CRC cell lines

**Fig. 1:**
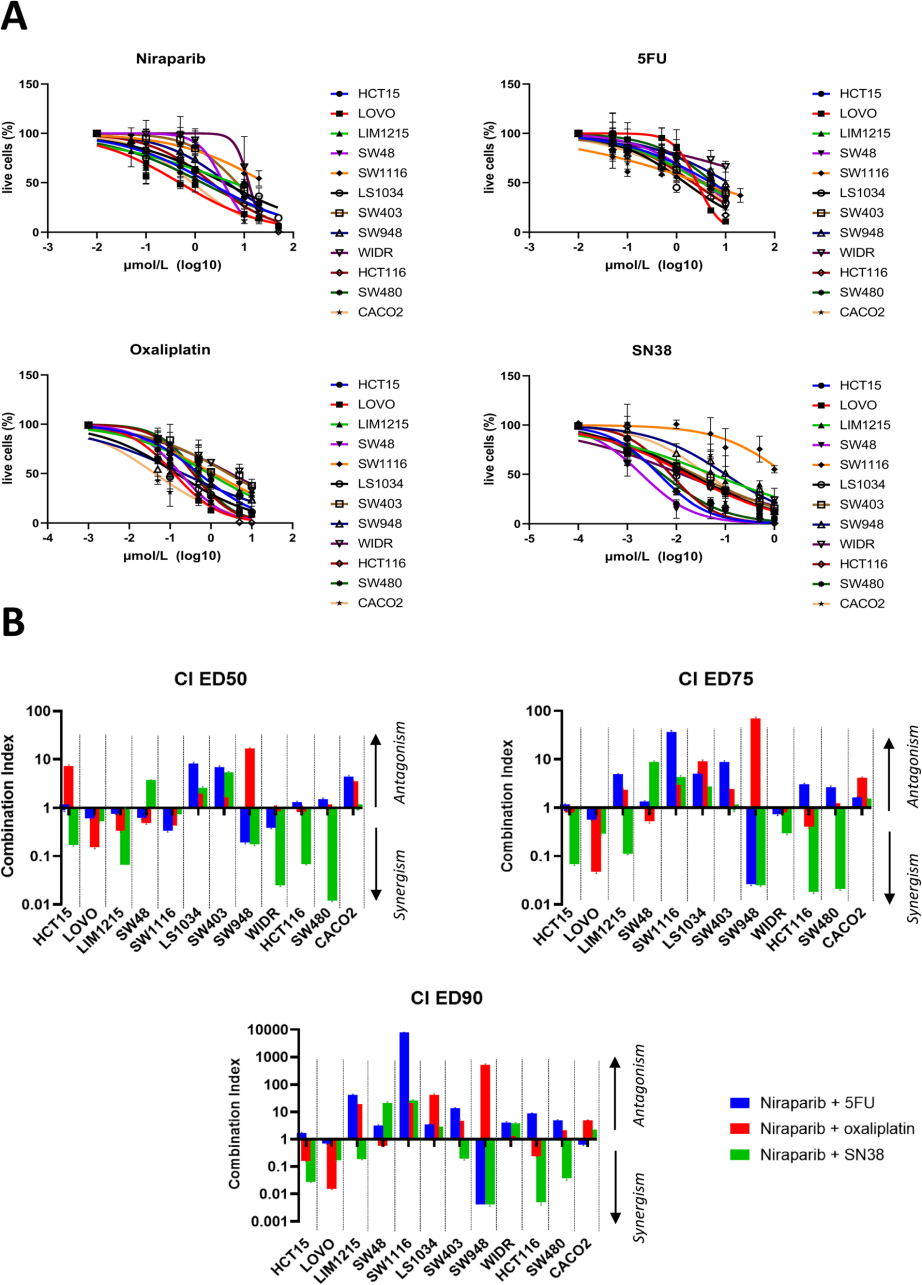
Activity of single agent 5-fluorouracil, oxaliplatin, SN38, and niraparib and niraparib-chemotherapy combinations in a panel of human CRC cell lines. **a**: 96 h proliferation assay (MTT) for niraparib, 5FU, oxaliplatin, SN38 in each of the cell lines included in the panel. **b**: Combination index according to the Chou-Talalay model of drug interaction at effective dose (ED) 50, ED75 and ED90. The combination between niraparib and SN38 is the more frequently synergistic across EDs (6/12 cell lines), with a combination index (CI) < 0,75

Twelve different human colorectal cancer cell lines with different genetic and transcriptomic profiles were tested for sensitivity to niraparib, 5-fluorouracil, oxaliplatin and SN38 (active metabolite of irinotecan) using the MTT assay (Fig. 1a). In Table 1, IC_50_ values for each drug are shown in parallel with the molecular characteristics of the cell lines used for the study. Most of the cell lines are sensitive to the three chemotherapeutic agents at concentrations reached in the clinical setting [28], while the IC_50_ for niraparib is higher than the clinically relevant plasma concentration of 1–2 μmol/L [29].

**Table 1:** Molecular features of the human CRC cell lines in comparison with their sensitivity to niraparib, 5-fluorouracil oxaliplatin or SN38 monotherapy

					IC_**50**_ (μM)			
Cell line	KRAS/BRAF status	MSI status	ATM and BRCA1/2 status	CMS	Niraparib	5FU	Oxaliplatin	SN38
***HCT15***	KRAS mut	MSI	ATM mut BRCA2 mut	CMS1	*1.4*	*6.2*	*0.3*	*0.006*
***LOVO***	KRAS mut	MSI		CMS1	*1.6*	*2*	*0.1*	*0.01*
***LIM1215***	RAS/BRAF wt	MSI	ATM mut	CMS1	*2.3*	*5.1*	*0.8*	*0.005*
***SW48***	RAS/BRAF wt	MSI	BRCA2 mut	CMS1	*4.3*	*5.3*	*0.2*	*0.001*
***SW1116***	KRAS mut	MSS		CMS2	*> 10*	*2.7*	*1*	*> 1*
***LS1034***	KRAS mut	MSS		CMS2	*6.2*	*1.5*	*0.1*	*0.02*
***SW403***	KRAS mut	MSS		CMS2	*6.3*	*3.7*	*2.7*	*0.008*
***SW948***	KRAS mut	MSS	ATM mut	CMS3	*2.8*	*14.5*	*0.1*	*0.06*
***WiDr***	BRAF mut	MSS		CMS3	*8.5*	*32*	*0.8*	*0.003*
***HCT116***	KRAS mut	MSI	ATM mut	CMS4	*1.4*	*2.2*	*0.25*	*0.005*
***SW480***	KRAS mut	MSS	ATM mut	CMS4	*2.1*	*5.4*	*0.4*	*0.005*
***CACO2***	RAS/BRAF wt	MSS		CMS4	*8.6*	*2.2*	*0.1*	*0.01*

*MSI* Microsatellite instable, *MSS* Microsatellite stable, *CMS* Consensus molecular subtype, *wt* wild type, *mut* Mutant. For ATM and BRCA2, only mutations predicted to be pathogenic are included

The effect of the combination of niraparib with each of the chemotherapeutics was calculated using the Chou-Talalay model. The combination of niraparib and SN38 showed the highest degree of synergism (combination index < 0.75) across all effective doses (EDs) in 6/12 CRC cell lines (HCT15, LOVO, LIM1215, SW948, HCT116, SW480). On the other hand, the combinations with 5-fluorouracil or oxaliplatin are mostly additive with significant synergism in only 1/12 (SW948) and 2/12 (LOVO, SW48) cell lines, respectively (Fig. 1b, supplementary Table 1).

**Fig. 2:**
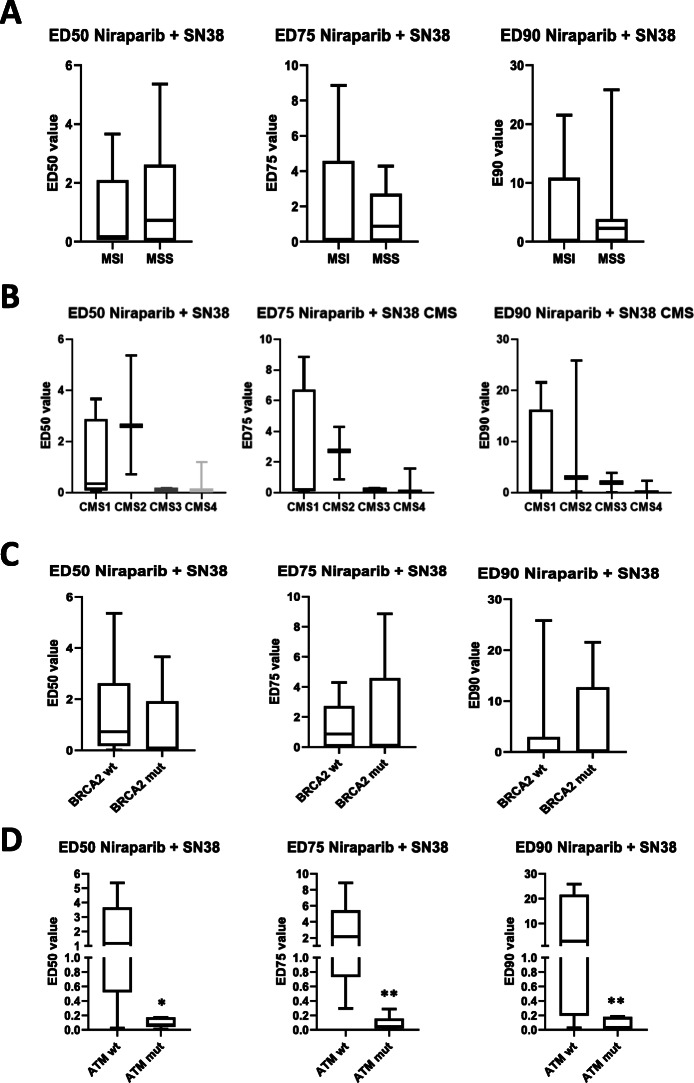
Correlation between molecular characteristics of the cell lines and synergism of the niraparib-SN38 combination. The distribution of the combination indexes (CIs) at ED50, ED75 and ED90 between MSS and MSI cell lines is not significantly different (**a**). No significant difference is also observed across consensus molecular subtypes (CMSs) (**b**). Cell lines carrying non-synonymous mutations in BRCA2 (BRCA2 mut) do not show a lower CI to the combination of niraparib and SN38 compared to BRCA2 wild type (wt) (**c**). On the other hand, cell lines carrying a non-synonymous or truncating mutation in ATM gene (ATM mut) present a significantly lower CI values with respect to ATM wild type (ATM wt) cell lines (**d**). *: *p* < 0.05; **: *p* < 0.01

Since genetic defects in HRR have been correlated to sensitivity to PARP inhibitors, we analyzed the mutational profiles for 29 relevant genes involved in homologous recombination for each cell line using the Cancer Cell Line Encyclopedia (CCLE) database (supplementary Table 2). ATM and BRCA2 constitute the two HRR genes with the highest recurrence of non-synonymous mutations in our panel (Table 1). We did not find any significant correlation between niraparib sensitivity and either microsatellite status, consensus molecular subtype (CMS), presence of non-synonymous ATM or BRCA2 mutations (Supplementary figure 1 A-D). Moreover, combination index values for the niraparib-SN38 combination showed no correlation with microsatellite status, CMS or pathogenic BRCA2 mutations (Fig. 2a-c).

Conversely, non-synonymous ATM mutations are present in 5/6 synergistic cell lines and significantly associated with synergism across three effective doses (ED50, ED75, ED90) (Fig. 2d). Notably, LOVO cell line, the only synergistic cell line that does not present a mutation in ATM, harbors an inactivating mutation in CHEK2, that represents one of the main downstream targets of ATM. The functional significance of ATM mutations in the cell panel investigated using the FATHMM bioinformatic tool is shown in Table 2, while the functional prediction of BRCA2 non-synonymous mutations was obtained using both the FATHMM tool and the Leiden Open Variation Database (LOVD) (Supplementary Table 3). These analyses show that all ATM mutations in our cell panel are functionally relevant, compared to BRCA2 mutations that are mostly neutral, with the exception of HCT15 and SW48 cell lines (Supplementary Table 3).

**Table 2:** Functional prediction according to FATHMM algorithm for COSMIC-identified non-synonymous, frameshift or truncating mutations of ATM in our panel

Cell lines	Genetic alteration ATM	COSMIC MUTATION ID	FATHMM prediction
**HCT15**	**c.1758G > T** **(p.E586D, missense)**	**COSV53760935**	**0.83 (pathogenic)**
**LIM1215**	**c.5557G > A** **(p.D1853N, missense)**	**COSV53728020**	**0.98 (pathogenic)**
**SW948**	**c.6628C > T** (p.Q2210*, nonsense)	**COSV53752944**	**0.97 (pathogenic)**
**HCT116**	**c.3380C > T** **(p.A1127V, missense)**	**COSV53735933**	**0.72 (pathogenic)**
**SW480**	**c.7382G > C** **(p.R2461P, missense)**	**COSV53782078**	**0.98 (pathogenic)**

### Preclinical validation of the combination of niraparib and irinotecan at low doses

#### Colony assay

The main limitation to the clinical development of PARP inhibitors/chemotherapy combinations is represented by the increased hematological toxicity, and the ideal dose ratio and schedule for a combination treatment is not known. For this reason, we tested the performance of the niraparib and SN38 combination compared to the single agents using different dose levels and concentration ratios (100:1 and 50:1), close to the ratio between the IC_50_ values for each single drug, with the aim to demonstrate whether low concentrations of both agents could retain a meaningful activity in our panel of cell lines.

**Fig. 3:**
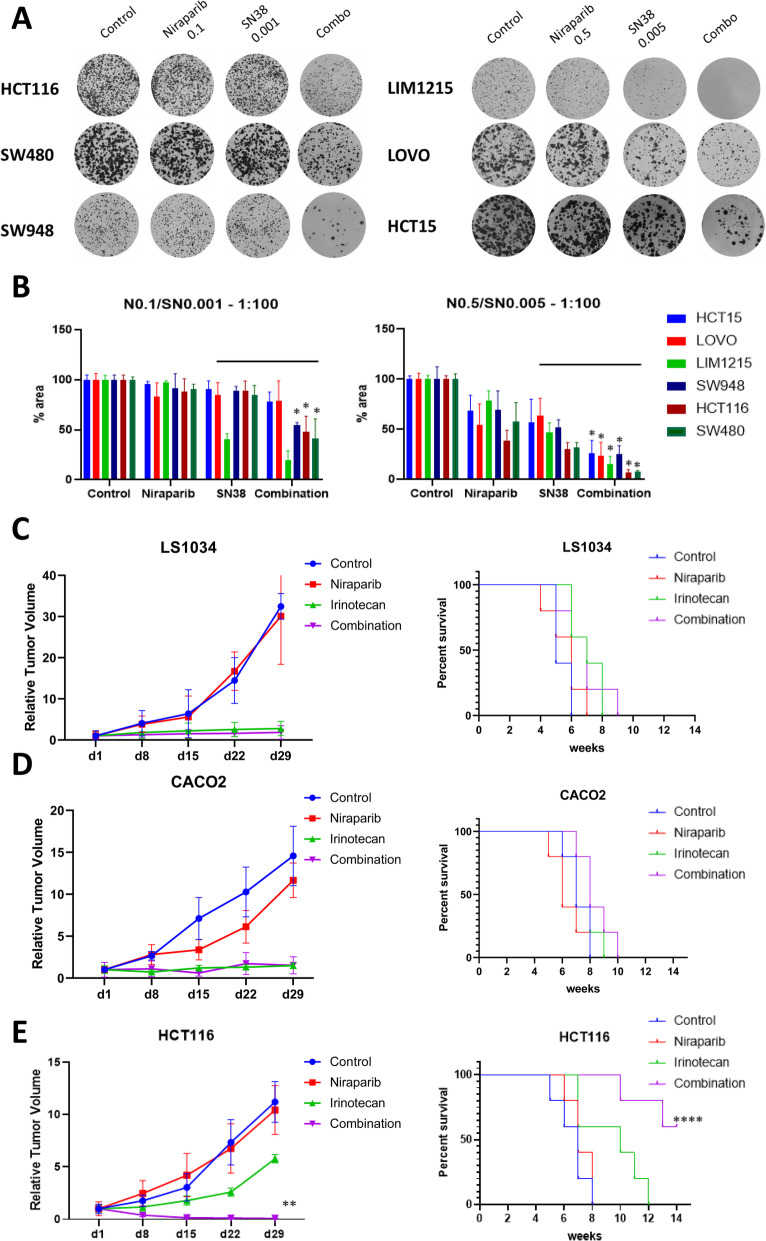
Colony assays and mice xenografts for niraparib-SN38 combination at low doses. **a**: Colony assays at indicated doses for synergistic cell lines. **b**: Bar graph representing surface area (normalized on control) for each dose ratio across the panel. Each bar corresponds to the mean of at least three experiments performed in duplicate. Two-way ANOVA was performed between SN38 and combination treatments. *: significant difference *p* < 0.05. **c-e**: Low dose combination treatment with niraparib (50 mg/kg p.o. d1–5 weekly) and irinotecan (10 mg/kg i.p. d2,d4 weekly) in LS1034, CACO2 and HCT116 xenograft models in nude mice. Relative tumor volumes (RTV) for control, niraparib, irinotecan, and combination arms are represented on left; log-rank survival analysis is represented on right. Combination treatment induces a significant decrease in tumor growth and statistically significant increased survival compared to irinotecan treatment only in HCT116 mouse xenograft. *: *p* < 0.05, **: *p* < 0.01, ****: *p* < 0.0001

Considering the cell lines in which the combination was synergistic, at the lowest doses (100 nM niraparib + 1 nM SN38) 3 out of 6 cell lines (SW948, HCT116 and SW480) exhibited significant colony growth inhibition with the combination compared to each drug alone, whereas at the following dose level (500 nM niraparib + 5 nM SN38) all of these 6 cell lines showed significant difference in colony formation when challenged with combination treatment (HCT15, LOVO, LIM1215, SW948, HCT116 and SW480) **(**Fig. 3a-b**)**.

Interestingly, at this dose level also WiDr cell line exhibited significant decrease in colony formation compared to each single drug, coherently with the evidence that the combination index values show synergism only at lower effective doses (ED50 and ED75) but not at ED90, while for the other cell lines (SW48, SW1116, LS1034, SW403, CACO2) the combination is not synergistic at these dose levels **(**Fig. 1b, Supplementary figure 2A).

Combination treatment is also effective at 50:1 concentration ratio (250 nM niraparib + 5 nM SN38) in all the synergistic cell lines, while using higher drug concentrations (2500 nM niraparib + 50 nM SN38) fails to elicit a synergistic response over SN38 alone, most likely due to high sensitivity to this agent in vitro, in both synergistic and non-synergistic cell lines (Supplementary figure 2 B-C).

#### Mice xenografts

To confirm the data obtained in vitro we established five mouse xenograft models using LS1034, Caco2, HCT116, SW48, and WiDr cell lines, that present different mutational profiles with regard to ATM and BRCA2. The niraparib dose used (50 mg/kg po d1–5 weekly) corresponds to the lowest concentration used in the in vitro experiments (100 nM for niraparib) and parallels the lowest active dose in human trials (30 mg/die) [29]. The dose used for irinotecan (10 mg/kg i.p. twice weekly) correspond to the lower doses used in human trials and is associated to similar SN38 plasma levels [30–32].

At these doses, consistently with in vitro experiments, in the absence of ATM mutations (LS1034) or in presence of ATM amplification (CaCo2), the combination did not show increased antitumor effect or survival advantage compared to irinotecan monotherapy (Fig. 3c-d). On the other hand, the ATM-mutated HCT116 tumor xenografts were significantly reduced by the combination compared to niraparib or irinotecan monotherapy. Notably, combination therapy induced complete tumor regression in 3/5 mice, and was coupled with significant increase in survival (3 mice still disease-free at the end of the 100 days follow up period) (Fig. 3e). In the absence of ATM mutations, the presence of BRCA2 mutations (pathogenic and non-pathogenic in SW48 and WiDr, respectively), did not confer advantage to the combination treatment (Supplementary figure 2B**)**.

The tolerability of the drugs given as single agents or in combination was good, with no significant body weight loss observed across treatment arms in mice (data not shown).

### Niraparib potentiate SN38 effect on double strand breaks and Rad51 recruitment

**Fig. 4:**
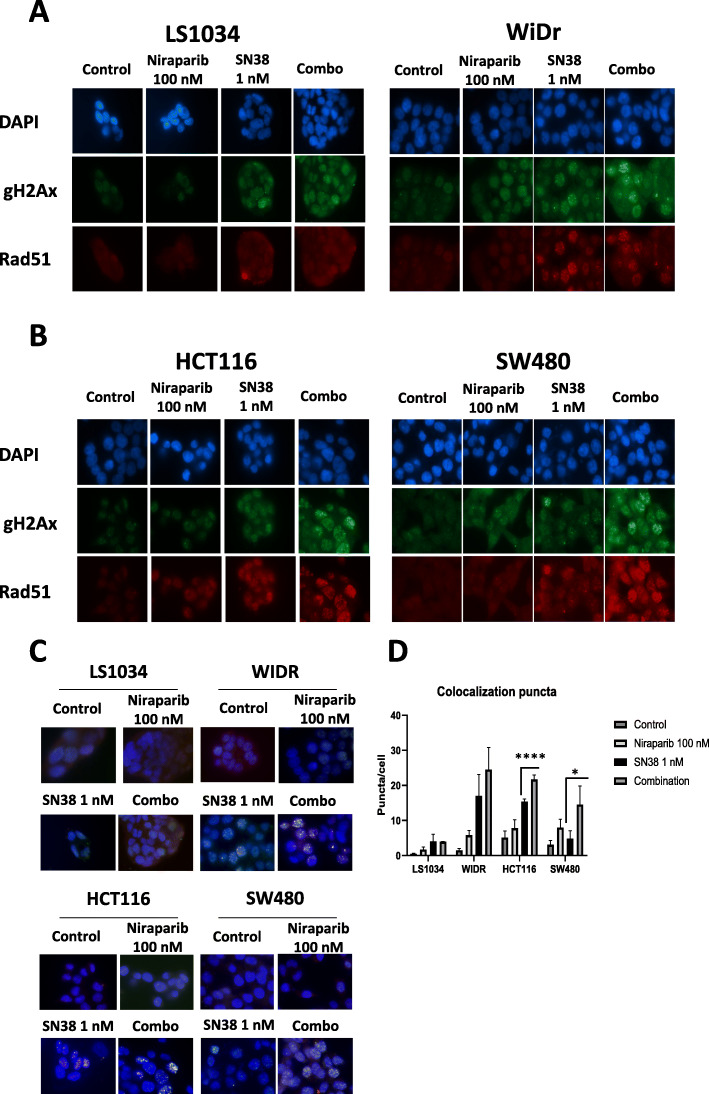
Niraparib increases double strand breaks and Rad51 recruitment induced by irinotecan. Two non-synergistic (**a**) and two synergistic (**b**) cell lines were immunostained for γH2Ax and Rad51 after 24 h incubation with low concentrations of the indicated agents (niraparib 100 nM, SN38 1 nM, or their combination). SN38 and combination treatment are able to induce DSBs marked by γH2Ax foci in all cell lines, while Rad51 foci are increased in synergistic cell lines (HCT116 and SW480) and in WIDR but are not increased in LS1034. **c**: co-localization of γH2Ax and Rad51 foci is increased in synergistic cell lines. **d**: γH2Ax and Rad51 colocalization foci are significantly increased with combination treatment compared to SN38 single agent in synergistic HCT116 and SW480 cell lines. *: *p* < 0.05, ****: *p* < 0.0001

It is well established that topoisomerase 1 inhibitors such as SN38 damage DNA by inducing single strand breaks (SSBs) that, if left unrepaired, can generate double strand breaks (DSBs) [14]. Since PARP inhibitors interfere with the mechanisms of SSB repair, we investigated whether the synergism between irinotecan and niraparib was related to increased DSBs generation and persistence 24 h after treatment. Indeed, the number of unrepaired DSBs that are generated by SN38 is increased by the combination treatment with niraparib in all the cell lines, as marked by γH2Ax foci **(**Fig. 4a-b**)**. HRR constitutes a key repair mechanism recruited after treatment with SN38 or the combination of niraparib and SN38 in WiDr, SW480 and HCT116, but not in LS1034, as evidenced by immunostaining with anti-Rad51 antibody that is a well characterized marker and the final effector of homologous recombination **(**Fig. 4a-b**)**. The colocalization of γH2Ax and Rad51 foci 24 h after treatment release represent the recruiting of HRR machinery on persistent DSBs (Fig. 4c). Notably, colocalization foci are significantly increased after combination treatment compared to SN38 only in synergistic HCT116 and SW480 cell lines (Fig. 4d).

Collectively, these data evidence that niraparib increases SN38-induced DSBs and that a higher sensitivity to the drug combination is accompanied by persistent engagement of homologous recombination repair at the sites of DSBs 24 h after treatment washout, particularly in HCT116 and SW480 ATM-mutated cell lines.

### Effects of the treatment on cell cycle

Previous reports have evidenced an induction of G2-M arrest by the combination of SN38 and PARPi [14, 15]. We examined the effects of niraparib, SN38 and their combination at low concentration on cell cycle distribution in ATM mutated (HCT116) and ATM wild type (LS1034) cell models. G2/M arrest is only present in the HCT116 cell line that is synergistic to the low-dose combination, whereas neither the single drugs nor the combination induces a cell cycle arrest in LS1034 (Supplementary figure 3).

### The combination treatment is more effective on human primary CRC 3D cell culture (spheroids) derived from ATM-mutated tumors

In order to validate the clinical relevance of the combination of niraparib and irinotecan, we tested the effects of single agents and their combination in a series of patient-derived primary 3D cultures (spheroids) from primary or metastatic colorectal cancers whose molecular characteristics have been obtained using FoundationOne comprehensive genomic panel (F1Cdx).

**Fig. 5:**
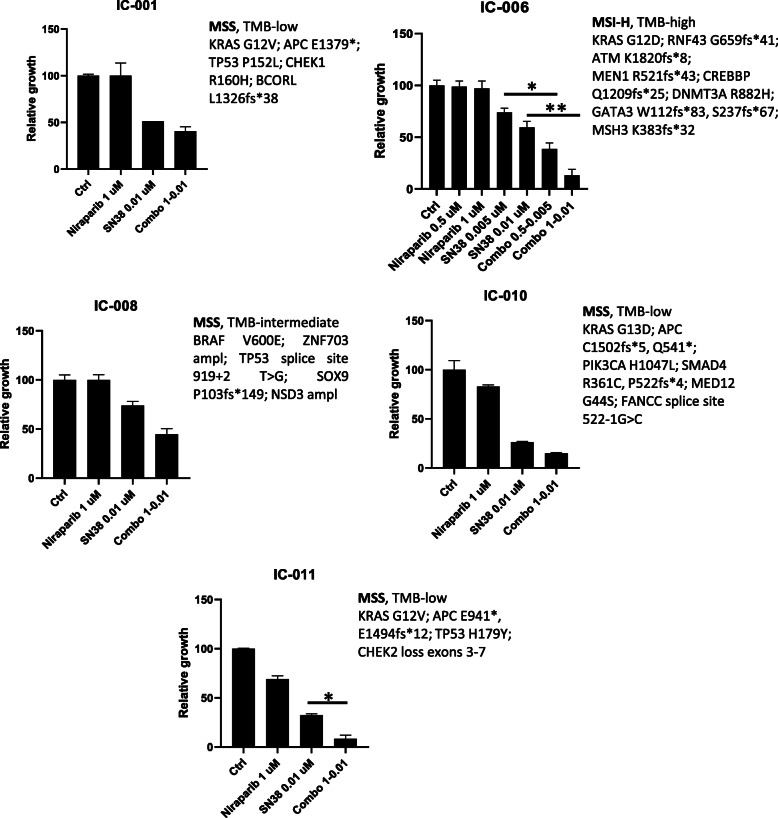
Ex vivo testing of niraparib, SN38, and their combination on human primary CRC 3D cell cultures (spheroids) according to the genetic background. Five primary 3D cell cultures (spheroids) were generated from surgical specimens (primary tumor or metastasis) of colorectal cancer. Tumor specimens were genetically profiled using a comprehensive genomic panel encompassing 324 frequently mutated cancer genes. Growth inhibition is significantly improved in IC-006 (ATM-mutated) at two concentration levels and in IC-011 (CHEK2-mutated). *p < 0.05; **: *p* < 0.01

All spheroids were treated with niraparib, SN38 and their combination at 100:1 dose ratio **(**Fig. 5)**.** The combination exhibited a significant stronger effect compared to single drugs in two out of five tested models, IC-006 and IC-011. In IC-006 model, derived from a tumor bearing an ATM mutation, the combination is effective at two dose levels (1:0.01 and 0.5–0.005), while in IC-011 it is only effective at the higher dose level (1:0.01). Notably, IC-011 spheroid derives from a tumor characterized by a CHEK2 inactivating mutation (Fig. 5). Interestingly, though not bearing the same mutation, CHEK2 is also mutated in LOVO cell line, the only cell line with significant synergism to the combination of niraparib and SN38 not presenting an ATM mutation.These data collectively confirm the in vitro and in vivo results, showing that the combination treatment with niraparib and irinotecan/SN38 is effective in a model of patient-derived 3D spheroids carrying an ATM mutation.

## Discussion

In our work, we have analyzed the activity of the combination between standard-of-care chemotherapeutics used in CRC and niraparib in a panel of 12 cell lines that encompasses the 4 consensus molecular subtypes, the different microsatellite status, and the different KRAS and BRAF mutational profiles. Niraparib was selected for this study due to its optimal pharmacodynamic and pharmacokinetic properties in patients, showing a strong PARP trapping activity (stronger than olaparib), but also a long half-life (> 24 h) and a wide therapeutic interval [29, 33]. Niraparib in vitro sensitivity was in line with previous reports in CRC cell lines, with mean IC_50_ values above the trough plasma concentration of this drug at the commonly used dose of 300 mg/die in monotherapy trials (Table 1**,** Fig. 1a), showing no association with microsatellite status (Supplementary figure 1) [6, 16, 29]. Moreover, transcriptional classification using the CMS classifiers did not evidence any correlation with sensitivity to the PARPi (Supplementary figure 1). SN38, the active metabolite of irinotecan, was shown to be the best partner for combination with niraparib, and the sensitivity to this combination was independent from microsatellite status or CMS (Fig. 1b, Supplementary Table 1, Fig. 2). Mutational profiles in 29 relevant genes in the HRR pathway were obtained for the cell lines included in the panel from public databases and were correlated with sensitivity to niraparib or the niraparib/SN38 combination. Notably, ATM and BRCA2, that constitute the most frequently mutated HR genes in large mCRC patients datasets such as MSKCC or COSMIC [19, 34], are also recurrently mutated in our panel (Table 1 and Supplementary Table 2). The functional prediction of these genetic alterations showed that in our cell panel all ATM mutations (5/5) were functionally relevant, compared with only 2/5 BRCA2-mutated cell lines carrying mutations with a pathogenic significance (Supplementary Table 3). However, in our panel, non-synonymous mutations in ATM or BRCA2 genes were not associated to a significant higher sensitivity to niraparib used as single agent (Supplementary figure 1). This may seem counterintuitive, as both ATM and BRCA2 inactivation are associated to homologous recombination deficiency and synthetic lethality with PARP inhibition [7], and a previous work demonstrated that ATM depletion using shRNAs induces sensitivity to PARP inhibitors [35]. However, it must be noted that a mutation in ATM does not correspond to ATM loss, though a decreased protein level was found in case of heterozygous ATM mutations in CRC cell lines compared to the wild type counterpart [35]. Nevertheless, we evidenced a significant association between the presence of ATM mutations and synergism with the niraparib-SN38 combination, while no correlation is present with microsatellite status, CMS or BRCA2 mutations (Fig. 2).

One possible explanation for the increased sensitivity to the combination in ATM-mutated cell lines lies in the relative insufficiency of timely DNA repair in these cells in case of double strand break damage overload. This dysfunction is unveiled by the combination treatment but not by single agent SN38, since most SN38-induced single strand breaks can be repaired before precipitating into DSBs [3]. Notably, both DSB recognition and HRR are intact in ATM-mutant cell lines, as evidenced by γH2Ax and Rad51 immunostaining (Fig. 4a-b), though persistent HR engagement upon combination treatment reflects a delay in the resolution of DSBs 24 h after treatment washout in case of heterozygous ATM mutations (Fig. 4c-d), possibly unveiling a haploinsufficient phenotype. The delay in repairing DSBs and the following cell cycle arrest are also evident at the cell cycle analysis in HCT116 compared to LS1034 cell lines (Supplementary Figure 3).

In this framework, the inhibition of both PARP and topoisomerase 1 is fundamental to achieve the effect in ATM-mutated CRCs, even if myelotoxicity characterizes a major concern that has hampered the development of other PARP inhibitors/chemotherapy combinations [17]. For this reason, we investigated whether the molecular subgroup characterized by the presence of pathogenic mutations in ATM exhibits an increased sensitivity to the niraparib/irinotecan combination at lower doses. Indeed, low drug concentrations of niraparib and SN38 induced a clonogenic arrest in vitro in synergistic cell lines (HCT15, LOVO, LIM1215, SW948, HCT116, SW480) (Fig. 3a-b). In mice models, low doses of niraparib and irinotecan were effective and synergistic in ATM-mutated HCT116 xenograft, in which they induced complete tumor regressions in 60% of treated mice (Fig. 3e). The low doses used for the mice experiments are predicted to achieve plasma concentrations of 100 nM for niraparib and less than 25 nM for SN38, concentrations that are way below the desirable ones in monotherapy, achieved with oral doses of 40 mg/die for niraparib and less than 100 mg/iv for irinotecan [29–32, 36].

Finally, we have also confirmed our findings in a relevant translational model using patient-derived 3D spheroids, in which the presence of a pathogenic mutation in ATM or in its downstream effector CHEK2 confer increased sensitivity to the combination of niraparib and SN38 (Fig. 5).

Recently, several papers have investigated the role of ATM mutations in cancer susceptibility and prognosis, suggesting that such mutations induce an attenuated cancer-predisposing phenotype in heterozygote carriers, that represent up to 1–2% of the general population [37], while presenting a favorable prognostic effect in CRCs bearing such heterozygous alterations [38]. Taken together, these evidences underpin a possible role for ATM heterozygous mutations in cancer therapy [39, 40]. Recently, a phase I study investigating different schedules for the combination of irinotecan and the PARP inhibitor rucaparib in refractory cancers bearing mutations in HRR genes was presented, showing how pulse schedules are clinically feasible and that the patient population with ATM-mutated cancers exhibits the greatest benefit from the combination [41].

## Conclusions

Our work shows that there is a molecularly defined subpopulation of CRCs bearing heterozygous mutations in ATM, accounting for up to 12% of patients [42], that may benefit from a combination treatment with niraparib and irinotecan used at low doses, suggesting a new potential approach in the treatment of colorectal cancer.

## Supplementary Information

**Additional file 1: Supplementary table 1.** Detailed values for combination index for each cell line and each niraparib + chemotherapeutics combination across 3 Effective Doses (EDs). ED50, ED75, and ED90 represent the required dose levels able to decrease cell viability to 50, 75, or 90%, respectively.

**Additional file 2: Supplementary table 2.** Mutational profiles HRR genes across the cell line panel. 29 genes (CHEK1, CHEK2, RAD51, BRCA1, BRCA2, BAP1, POLQ, ATM, ATR, MDC1, PARP1, FANCF, FANCM, BRIP1, FANCE, WRN, CDK12, MDC1, FAN1, NBN, FANCA, RAD51C, RAD51D, EXO1, RBBP8, FANCD2, NONO, SMC5, USP11) with relevance in the HRD phenotype (Riaz, Nat Commun 2017) were analyzed. Mutational profiles were obtained from cBio-portal using the Cancer Cell Line Encyclopaedia (CCLE) dataset [last accessed June 20th 2020].

**Additional file 3: Supplementary table 3.** BRCA2 mutations in the cell panel. Functional prediction according to FATHMM algorithm for COSMIC-identified non-synonymous, frameshift or truncating mutations of BRCA2 in our panel. Whenever available, Leiden Open Variant Database (LOVD) reference is also included [last accessed July 1st 2020].

**Additional file 4: Supplementary figure 1.** Niraparib IC_50_ values were correlated to microsatellite status (**A**), CMS classification (**B**), presence of genetic alterations in ATM (**C**), presence of genetic alterations in BRCA2 (**D**). No significant difference was evidenced, using the Mann-Whitney test. MSI: microsatellite instability; MSS: microsatellite stability; CMS: consensus molecular subtype; ATM wt: no mutations in ATM or CHEK2; ATM mut: mutations in ATM and/or CHEK2.

**Additional file 5: Supplementary figure 2.** Colony assays and mice xenografts for niraparib-SN38 combination in non-synergistic cell lines. A: Colony assays at indicated doses for non-synergistic cell lines. **B:** Bar graph representing surface area (normalized on control) for each dose ratio across the panel. Each bar corresponds to the mean of at least three experiments performed in duplicate. Two-way ANOVA was performed between SN38 and combination treatments. *: significant difference *p* < 0.05, no asterisk: non-significant difference. **C-D:** Low dose combination treatment with niraparib (50 mg/kg p.o. d1-5 weekly) and irinotecan (10 mg/kg i.p. d2,d4 weekly) in SW48 and WIDR xenograft models in nude mice. Relative tumor volumes (RTV) for control, niraparib, irinotecan, and combination arms are represented on left; log-rank survival analysis is represented on right.

**Additional file 6: Supplementary figure 3.** Effect of the treatment on cell cycle. A: Combination treatment is able to induce a G2M arrest in cell cycle only in HCT116 (synergistic cell line), while is ineffective in LS1034 (non-synergistic cell line). A sub-G0G1 peak is evidenced in synergistic HCT116 cells after combination treatment, possibly reflecting apoptosis.

## Data Availability

All data generated or analysed during this study are included in this published article and its supplementary information files.

## Abbreviations

CRC: Colorectal Cancer
mCRC: Metastatic Colorectal Cancer
MMR: Mismatch Repair
NER: Nucleotide Excision Repair
HRR: Homologous Recombination Repair
DDR: DNA Damage Response
PARPi: Poly-ADP Ribose Polymerase inhibitor
MSI: Microsatellite Instability
HRD: Homologous Recombination Deficiency
CMS: Consensus Molecular Subtype
CCLE: Cancer Cell Line Encyclopedia
COSMIC: Catalogue of Somatic Mutations in Cancer
FFPE: Formalin Fixed Paraffin Embedded
TMB: Tumor Mutational Burden
ANOVA: Analysis of Variance
5FU: 5-fluorouracil
ED: Effective dose
IC50: Inhibitory concentration 50
CI: Combination index
SSB: Single Strand Break
DSB: Double Strand Break
