# Supplementary material for: Vulnerability to low-dose combination of irinotecan and niraparib in ATM-mutated colorectal cancer
Source: J Exp Clin Cancer Res. 2021 Jan 6;40:15. doi: 10.1186/s13046-020-01811-8 (PMC7789007; doi:10.1186/s13046-020-01811-8)
Supplement: Supplementary file 1 — Additional file 1: Supplementary table 1. Detailed values for combination index for each cell line and each niraparib + chemotherapeutics combination across 3 Effective Doses (EDs). ED50, ED75, and ED90 represent the required dose levels able to decrease cell viability to 50, 75, or 90%, respectively. [file 13046_2020_1811_MOESM1_ESM.pdf]

Supplementary table 1

		HCT15	LOVO	LIM1215	SW48	SW1116	LS1034	SW403	SW948	WIDR	HCT116	SW480	CaCo2
ED50	Niraparib/5FU	0.885	0.602	0.764	0.619	0.333	8.209	6.935	**0.191**	0.390	1.334	1.521	4.431
ED50	Niraparib/oxaliplatin	7.311	**0.152**	0.333	**0.487**	0.427	1.977	1.663	16.740	0.837	0.834	0.888	3.496
ED50	Niraparib/SN38	**0.170**	**0.519**	**0.067**	3.662	**0.727**	2.615	5.362	**0.174**	**0.025**	**0.069**	**0.012**	1.189

		HCT15	LOVO	LIM1215	SW48	SW1116	LS1034	SW403	SW948	WIDR	HCT116	SW480	CaCo2
ED75	Niraparib/5FU	1.168	0.571	4.876	1.352	36.891	5.108	8.858	**0.026**	0.735	3.067	2.659	1.634
ED75	Niraparib/oxaliplatin	1.064	**0.047**	2.388	**0.522**	2.950	9.122	2.441	70.568	0.788	0.412	1.255	4.110
ED75	Niraparib/SN38	**0.068**	**0.287**	**0.112**	8.862	4.288	2.724	0.878	**0.025**	**0.297**	**0.018**	**0.021**	1.571

		HCT15	LOVO	LIM1215	SW48	SW1116	LS1034	SW403	SW948	WIDR	HCT116	SW480	CaCo2
ED90	Niraparib/5FU	1.716	0.692	41.093	3.186	8007.810	3.448	13.510	**0.004**	4.056	8.704	4.945	0.626
ED90	Niraparib/oxaliplatin	0.160	**0.015**	19.205	**0.567**	21.766	42.115	4.616	526.025	0.796	0.240	2.131	4.820
ED90	Niraparib/SN38	**0.028**	**0.166**	**0.188**	21.522	25.846	2.927	0.197	**0.004**	3.848	**0.005**	**0.036**	2.292
