# Supplementary material for: Vulnerability to low-dose combination of irinotecan and niraparib in ATM-mutated colorectal cancer
Source: J Exp Clin Cancer Res. 2021 Jan 6;40:15. doi: 10.1186/s13046-020-01811-8 (PMC7789007; doi:10.1186/s13046-020-01811-8)
Supplement: Supplementary file 3 — Additional file 3: Supplementary table 3. BRCA2 mutations in the cell panel. Functional prediction according to FATHMM algorithm for COSMIC-identified non-synonymous, frameshift or truncating mutations of BRCA2 in our panel. Whenever available, Leiden Open Variant Database (LOVD) reference is also included [last accessed July 1st 2020]. [file 13046_2020_1811_MOESM3_ESM.pdf]

Supplementary table 3

Cell lines	Genetic alteration BRCA2	COSMIC MUTATION ID	FATHMM prediction	LOVD reference
HCT15	c.5351dup (p.N1784T fs*7, frameshift)	COSV66448906	n/a	Pathogenic
	c.3599_3600del (p.C1200*, nonsense)	COSV66458816	n/a	Pathogenic
	c.8351G>A (R2784Q, missense)	n/a	n/a	VUS
	c.1460C>T (p.A487V, missense)	COSV66458810	0.05 (neutral)	n/a
LIM1215	c.7397T>C (p.V2466A, missense)	COSV66451785	0.00 (neutral)	Benign
SW48	c.5351dup (p.N1784T fs*7, frameshift)	COSV66448906	n/a	Pathogenic
	c.7397T>C (p.V2466A, missense)	COSV66451785	0.00 (neutral)	Benign
	c.5073del (p.K1691Nfs*15, frameshift)	COSV66451438	n/a	Pathogenic
	c.4258G>T (p.D1420Y, missense)	COSV66451253	0.50 (neutral)	Benign
WIDR	c.7397T>C (p.V2466A, missense)	COSV66451785	0.00 (neutral)	Benign
HCT116	c.8021dup (p.I2675Dfs*6, frameshift)	COSV66451450	n/a	VUS
