# Supplementary figures and images for: Vulnerability to low-dose combination of irinotecan and niraparib in ATM-mutated colorectal cancer
Source: J Exp Clin Cancer Res. 2021 Jan 6;40:15. doi: 10.1186/s13046-020-01811-8 (PMC7789007; doi:10.1186/s13046-020-01811-8)

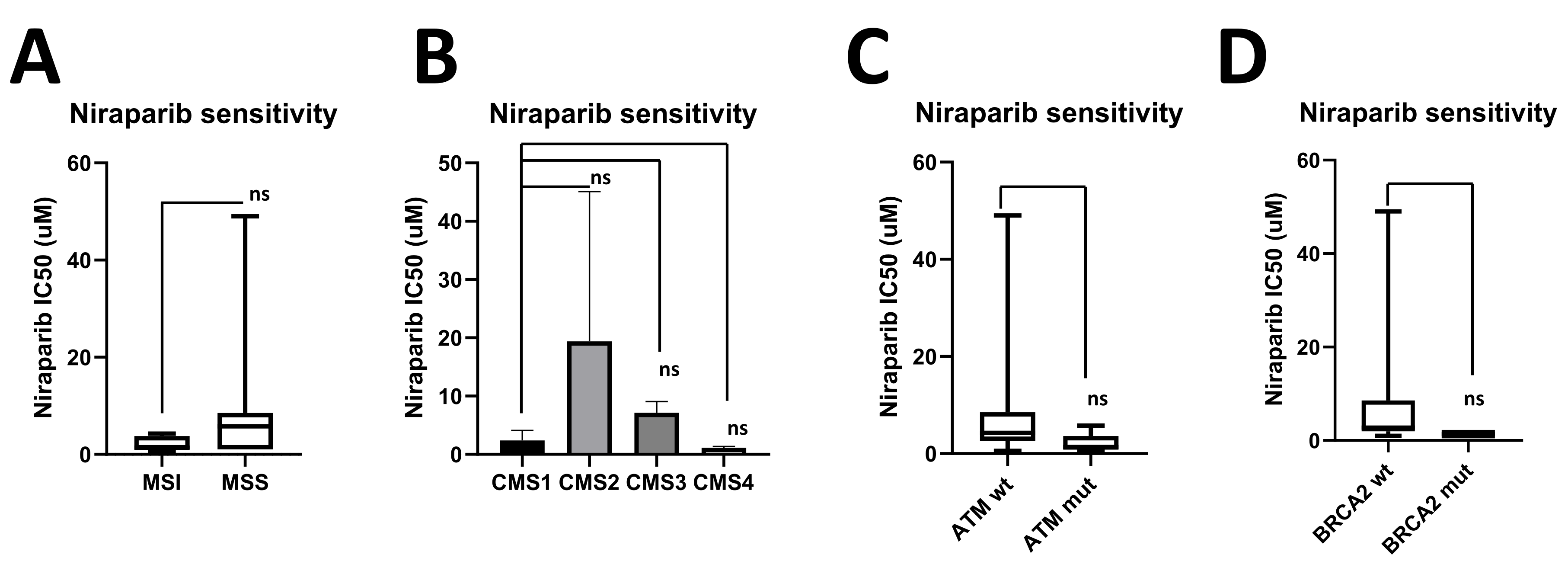

Supplement: Supplementary file 4 — Additional file 4: Supplementary figure 1. Niraparib IC50 values were correlated to microsatellite status (A), CMS classification (B), presence of genetic alterations in ATM (C), presence of genetic alterations in BRCA2 (D). No significant difference was evidenced, using the Mann-Whitney test. MSI: microsatellite instability; MSS: microsatellite stability; CMS: consensus molecular subtype; ATM wt: no mutations in ATM or CHEK2; ATM mut: mutations in ATM and/or CHEK2. [file 13046_2020_1811_MOESM4_ESM.png]

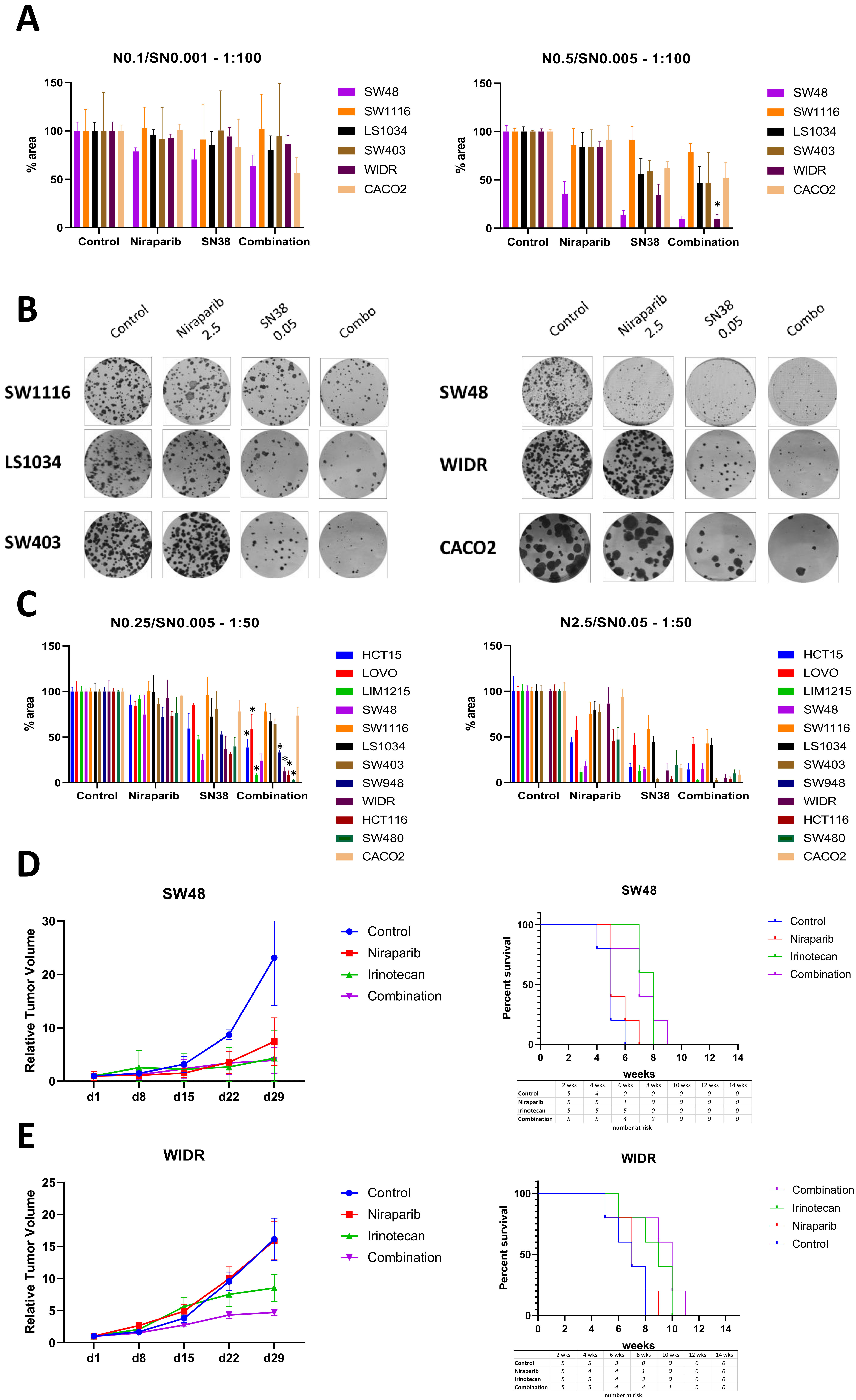

Supplement: Supplementary file 5 — Additional file 5: Supplementary figure 2. Colony assays and mice xenografts for niraparib-SN38 combination in non-synergistic cell lines. A: Colony assays at indicated doses for non-synergistic cell lines. B: Bar graph representing surface area (normalized on control) for each dose ratio across the panel. Each bar corresponds to the mean of at least three experiments performed in duplicate. Two-way ANOVA was performed between SN38 and combination treatments. *: significant difference p < 0.05, no asterisk: non-significant difference. C-D: Low dose combination treatment with niraparib (50 mg/kg p.o. d1-5 weekly) and irinotecan (10 mg/kg i.p. d2,d4 weekly) in SW48 and WIDR xenograft models in nude mice. Relative tumor volumes (RTV) for control, niraparib, irinotecan, and combination arms are represented on left; log-rank survival analysis is represented on right. [file 13046_2020_1811_MOESM5_ESM.png]

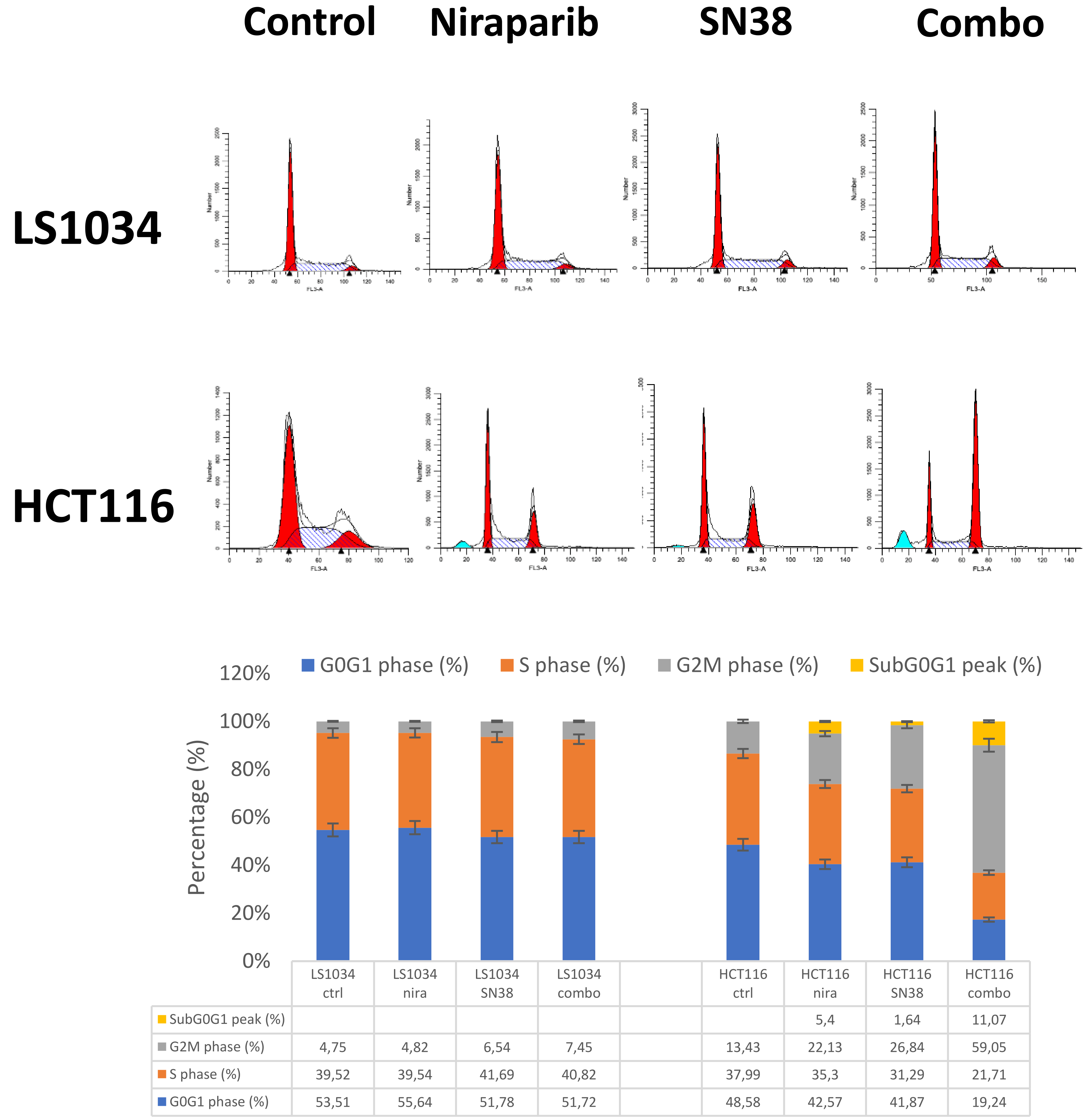

Supplement: Supplementary file 6 — Additional file 6: Supplementary figure 3. Effect of the treatment on cell cycle. A: Combination treatment is able to induce a G2M arrest in cell cycle only in HCT116 (synergistic cell line), while is ineffective in LS1034 (non-synergistic cell line). A sub-G0G1 peak is evidenced in synergistic HCT116 cells after combination treatment, possibly reflecting apoptosis. [file 13046_2020_1811_MOESM6_ESM.png]
